# Influence of Target Concentration and Background Binding on *In Vitro* Selection of Affinity Reagents

**DOI:** 10.1371/journal.pone.0043940

**Published:** 2012-08-28

**Authors:** Jinpeng Wang, Joseph F. Rudzinski, Qiang Gong, H. Tom Soh, Paul J. Atzberger

**Affiliations:** 1 Department of Mechanical Engineering, University of California Santa Barbara, Santa Barbara, California, United States of America; 2 Department of Mathematics, University of California Santa Barbara, Santa Barbara, California, United States of America; 3 Materials Department, University of California Santa Barbara, Santa Barbara, California, United States of America; Indian Institute of Science, India

## Abstract

Nucleic acid-based aptamers possess many useful features that make them a promising alternative to antibodies and other affinity reagents, including well-established chemical synthesis, reversible folding, thermal stability and low cost. However, the selection process typically used to generate aptamers (SELEX) often requires significant resources and can fail to yield aptamers with sufficient affinity and specificity. A number of seminal theoretical models and numerical simulations have been reported in the literature offering insights into experimental factors that govern the effectiveness of the selection process. Though useful, these previous models have not considered the full spectrum of experimental factors or the potential impact of tuning these parameters at each round over the course of a multi-round selection process. We have developed an improved mathematical model to address this important question, and report that both target concentration and the degree of non-specific background binding are critical determinants of SELEX efficiency. Although smaller target concentrations should theoretically offer superior selection outcome, we show that the level of background binding dramatically affect the target concentration that will yield maximum enrichment at each round of selection. Thus, our model enables experimentalists to determine appropriate target concentrations as a means for protocol optimization. Finally, we perform a comparative analysis of two different selection methods over multiple rounds of selection, and show that methods with inherently lower background binding offer dramatic advantages in selection efficiency.

## Introduction

**Figure 1:**
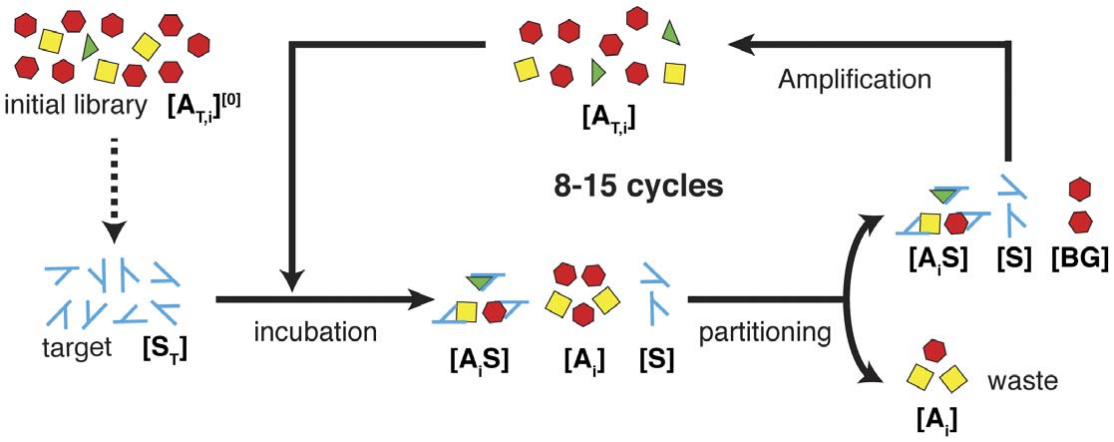
SELEX scheme for selecting high affinity aptamers. The target molecules and aptamer library are incubated and then partitioned to separate unbound and target-bound aptamers. Selected aptamers are amplified via polymerase chain reaction (PCR) to create an enriched pool for use in the next round of selection. Typically, 8–15 rounds of selection are required to isolate aptamers with high affinities. In our model, [*A_i_*] denotes the concentration of unbound aptamers of type *i* and [*S*] denotes the concentration of unbound target molecules. The concentration of target-bound aptamers is denoted by [*A_i_S*] for aptamers of type *i*.

Relative to other commonly used affinity reagents, nucleic acid-based aptamers possess many useful features including chemical synthesis, reversible folding, thermal stability and low cost, making them a promising alternative to antibodies and other protein-based reagents [1]–[3]. To date, DNA or RNA aptamers have been generated for a wide variety of molecular targets including proteins [4], small molecules [5], cell surfaces [6], [7], and even whole organisms [8]. Aptamers are typically isolated from combinatorial oligonucleotides libraries via a method of selection called Systematic Evolution of Ligands by EXponential enrichment (SELEX), which entails an iterative process of binding, separation and amplification (**Fig. 1**) [9], [10]. In SELEX, a large population of nucleic acid molecules is chemically synthesized wherein each molecule contains random sequences that are able to adopt unique conformations through intramolecular binding. Candidate molecules are selected for their ability to specifically bind to a chosen target, and selected molecules are amplified to create more copies. The cycle of selection and amplification is repeated to successively enrich aptamers with high affinities. Though conceptually simple, SELEX is time-consuming and resource-intensive and does not always yield reagents with desired characteristics. For example, when one tabulates the affinity of DNA aptamers for protein targets in literature, one observes a large variability spanning six orders of magnitude [11]. Multiple factors contribute to this large variability, including the structure and charge state of the target, design and complexity of the library, as well as a variety of experimental factors in selection and measurement.

To gain insights into those experimental factors that can influence SELEX, a number of investigators have developed theoretical models and performed numerical simulations of the selection process [12]–[17]. For example, seminal work by Irvine, Tuerk and Gold performed an extensive mathematical analysis of selection to investigate how experimental conditions such as target concentration, background binding, and partitioning efficiency of high-affinity aptamers can affect resulting aptamers [12], [14]. Building on these results, Levine and Nilsen-Hamilton provided sufficient conditions and related theorems to show the circumstances under which selection is ensured to converge to the optimal molecule within the library [15]. Furthermore, to investigate the role of the discrete number of molecules undergoing selection, Waterman and coworkers developed a probabilistic model to study the link between the number of target molecules and the number of PCR amplification cycles performed on the probability of achieving convergence to the best molecule within the library [18]. However, the selection conditions in previous work were considered to be static over multiple rounds of selection, and the impact of their continuous optimization - at each round - have not been previously considered.

To address this important issue, we have performed a mathematical analysis of the critical experimental conditions that can influence the affinity distribution of the selected aptamer pool. Based on these data, we developed a model that uses the binding characteristics of a given library or aptamer pool and the non-specific background binding level associated with particular SELEX conditions to determine the ideal target concentration for optimal selection of high affinity aptamers at each selection round. We also used our model to compare two different SELEX methods, with low and high levels of background binding. We show that under low-background conditions, high selection stringency can be applied to achieve rapid convergence of the library to the highest affinity aptamer, whereas the high-background method limits the maximal enrichment at each round, requiring more selection rounds to achieve convergence. Interestingly, enrichment in the low-background method depends less sensitively on target concentration to achieve optimal enrichment. In contrast, the greater sensitivity associated with the high-background method means that a considerably narrower range of target concentration is required to attain efficient selection, necessitating tighter control of experimental conditions.

## Methods

### Mathematical Model for SELEX

#### Chemical kinetics

In our notation, [*A_i_*] denotes the concentration of unbound aptamers of type *i* and [*S*] denotes the concentration of unbound target molecules. The concentration of target-bound aptamers is denoted by [*A_i_S*] for aptamers of type *i*, as shown in Figure 1.

The binding and unbinding kinetics are modeled by
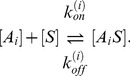
(1)



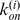
 denotes the association rate for an aptamer binding available free target molecules, and 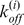 denotes the dissociation rate for that aptamer from the target molecule. In our analysis, we assume a Langmuirian 1∶1 interaction, in which only one aptamer molecule may bind to a single target molecule.

The rate of change of the concentration of aptamer-target complexes is given by
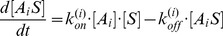
(2)


The total concentration of target is given by
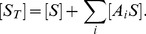
(3)


The total concentration of aptamers of type *i*, both bound and unbound, is given by
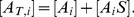
(4)


The total target concentration given in equation (3) and the total aptamer concentration given in equation (4) are conserved quantities that remain constant throughout each round of selection. The aptamer-target dissociation constant for aptamers of type *i* is denoted by
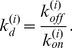
(5)


#### Steady-state after incubation step

Each selection step of SELEX entails the collection of aptamers from the starting library that are bound to target molecules. To model this aspect of SELEX and the population of bound aptamers, we assume that the reactions of the system during this incubation step reach equilibrium. In this case, the concentration of free and bound aptamers is given by the steady-state of the kinetic equation (2). Under these assumptions, and following an approach similar to [15], an implicit equation for the steady-state concentrations can be obtained in terms of unbound free target as
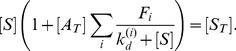
(6)


In equation (6), the fraction of type *i* aptamers in the library is denoted by
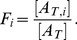
(7)


From the steady-state of equation (2), the dissociation constant of type *i* aptamers can be expressed as
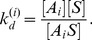
(8)


The target bound aptamer concentration can be expressed as
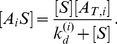
(9)


This is obtained by letting equation (2) be equal to zero and using equation (4) and (5).

To characterize this population, it is useful to define a bulk equilibrium dissociation constant 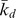
for the pool of aptamers, similar to the conventional definition of the dissociation constant 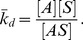
(10)


[*A*] denotes the concentration of unbound free aptamers and [*AS*] denotes the total concentration of bound aptamers irrespective of type. This allows for the bound aptamer concentration to be expressed as
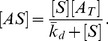
(11)where
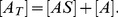
(12)


#### Model for selection without background binding

Each round of SELEX produces a new aptamer pool. In the ideal case, in which only aptamers specifically bound to target are recovered, the new pool of aptamers is described based on our analysis above by
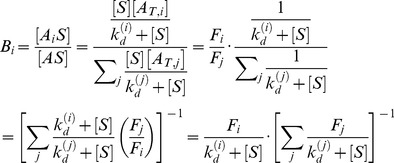
(13)


*B_i_* denotes the fraction of type *i* aptamers in the newly selected aptamer pool. *F_i_* denotes the fraction of type *i* aptamers in the initial library subject to selection, as defined in equation (7). By using the bulk dissociation constant of the library 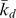, defined by equation (10), and combining equations (9) and (10), the fraction of type *i* aptamers *B_i_* can be simplified and expressed as
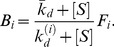
(14)


In equation (14), it is important to note that *F_i_*, and 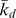 refers to the aptamer population prior to the current round of selection, however, *B_i_* refers to the fraction of bound type *i* aptamers after the round. We denote the round of selection by the superscript *n*. With this convention for indexing, the successive aptamer distributions after the (n+1)^th^ round of selection can be expressed as
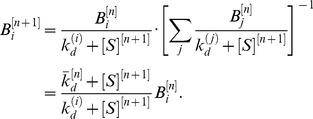
(15)


From equation (15), 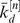 is given by
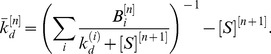
(16)


We remark that after a single round of selection, aptamers of type *i* with dissociation constants larger than 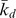 will comprise a smaller fraction of the new pool (since the prefactor multiplying *F_i_* will be less than one) and those with dissociation constants smaller than 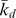 will be enriched and comprise a greater fraction of the new pool (since the prefactor multiplying *F_i_* will be greater than one). The target concentration is an important experimental parameter that influences the selection outcome. Within experimentally feasible range, a smaller target concentration should yield a smaller unbound free target concentration [*S*].

#### Model for selection with background binding

In the above analysis, we assumed that all aptamers not bound to target are removed during the partitioning step of SELEX. However, in practice, the partitioning process is not ideal, and a fraction of unbound aptamers can be non-specifically retained during the partitioning step and will be present in the selected pool. These constitute the background aptamers (BA). To model such experimental realities more accurately, we consider the effect of BA on the SELEX process. 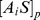 denotes the concentration of aptamers of type *i* that are captured during the partitioning step. Since the volume remains constant during the same round, we model background contributions to partitioning by



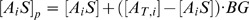
(17)


BG is the fraction of BA of type *i* that are recovered during partitioning. We remark that 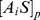 includes both the aptamers specifically bound to targets as well as the BA. The fraction of type *i* aptamers that appear in the new pool after a round of selection is given by
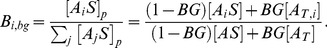
(18)


This can be expressed as
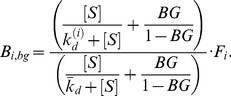
(19)


This follows by substituting equations (7) and (9) into equation (18). This shows clearly how the fraction of aptamers of type *i* obtained in the new pool is influenced by background binding during selection. We remark that in the case where the limit for BG tends to zero, we recover the expression for pure selection without background (see equation (14)). In the case where the limit of BG tends to one, we find that background binding results in loss of selective pressure, and the fraction of aptamers of type *i* selected in the new pool is equal to the fraction of aptamers of type *i* in the previous pool. We note that the population of low affinity aptamers resulting from unintended binding to experimental apparatus (e.g. filters, beads, or other solid support) can also be treated as non-specific background binders. In this case, it may be appropriate to model the phenomenon using a multi-target SELEX model [14], [17].

#### Model of the affinity distribution in the initial random library

Aptamer selection begins with a large random combinatorial library of nucleic acids, typically containing up to ∼10^14^ molecules. These molecules are expected to have a wide range of affinities to a given target molecule, depending on the specific molecular interactions between an individual aptamer and that target. Important factors contributing to this affinity include aptamer/target charge state, size, structural stability and distribution of cationic/anionic regions [11]. For the library as a whole, it is expected that many different aptamers will have similar affinities for a target. We shall model the library by considering it as a collection of sub-types, with each aptamer grouped into a class according to its affinity as characterised by its equilibrium dissociation constant (*k_d_*).

**Figure 2:**
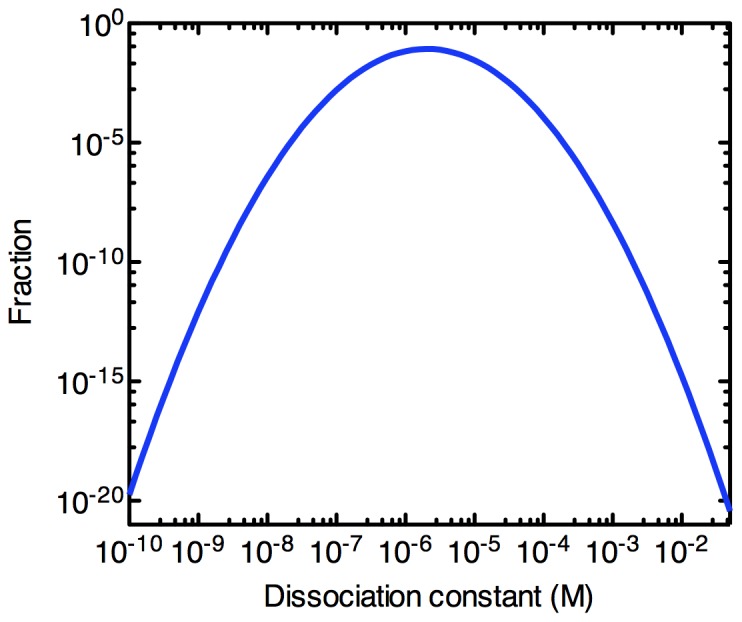
Affinity distribution of the initial random library. To model this log-normal distribution, we used a normal probability density function with μ = −13.1 and σ = 1.07, yielding a bulk *k_d_* of 1.25 µM.

To investigate the *in vitro* selection of aptamers, it is necessary to consider the initial affinity distribution of the combinatorial library for a target of interest. It is commonly hypothesized that the binding free energies (*ΔG*) between nucleic acids and proteins are normally distributed [13], [19]. The binding free energy is related to the equilibrium dissociation constant (*k_d_*) by



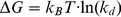
(20)where *k_B_* is the Boltzmann constant and *T* is the absolute temperature. This makes it natural to consider a log-normal distribution for the equilibrium dissociation constants (*k_d_*) of library components. The affinity distribution of library molecules for the target protein has the probability density function



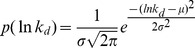
(21)To model this log-normal distribution, we use the normal probability density function with μ and σ be −13.1 and 1.07 respectively. This affinity distribution, shown in Figure 2, is consistent with the reported value in the literature [12], [13], where the bulk *k_d_* is in the low µM range. For our model, we arbitrarily chose a bulk 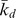 value of 1.25 µM and this affinity distribution is used throughout this work. Furthermore, we define high affinity aptamers (HAA) as those possessing *k_d_*<1 nM to the target molecule.

#### Computational methods

We modeled the SELEX process by solving a system of non-linear equations having a dimension comparable to the number of different types of aptamers (see equation (9)). To gain quantitative and qualitative insights into the SELEX process, we used numerical methods to compute solutions. Our analysis shows that this high dimensional problem can in fact be reduced to solving only one non-linear equation, which contains only one unknown, the unbound free target concentration [*S*] (see equation (6)). This simplification will be employed throughout our simulation studies. We used Newton iterations to find the solution for [*S*]. All of the other concentrations associated with this model can be calculated from [*S*] by using equations (3), (4) and (9). By using equation (13), this procedure yields an efficient method to determine the affinity distribution of the new aptamer pool after each round of selection. The bulk dissociation constant 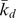 can also be readily calculated by using equation (16).

## Results

### Role of Target Concentration without Background Binding

To analyze the efficiency of each round of selection, we defined the enrichment of aptamers of type *i* as
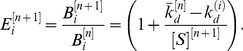
(22)


The superscript *n* denotes the round of selection being considered. The enrichment describes the change in the affinity distribution of aptamers of type *i* between successive rounds of selection.

**Figure 3:**
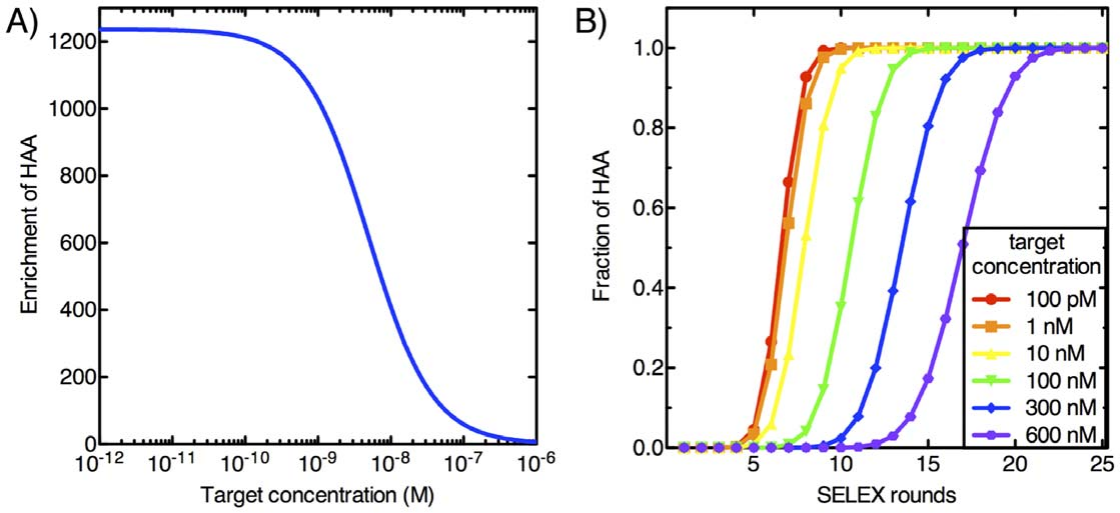
Influence of target concentration on selection efficiency. (A) Enrichment of HAA as a function of target concentration. As the target concentration is varied over 6 orders of magnitude, from 1 µM to 1 pM, the enrichment of HAA increases from 6.6 to 1240, respectively. (B) The fraction of HAA in the selected pool as a function of selection rounds for varying target concentrations.

The target molecule concentration used during the selection process is an important parameter that governs selection pressure. The above model predicts that as the amount of target decreases, the level of HAA enrichment will increase. This suggests that enrichment is greatest when using the lowest feasible target concentration. To investigate the role of target concentration in enrichment and convergence of SELEX, we varied the target concentration over six orders of magnitude, from 1 µM to 1 pM. We show a simulation of enrichment of HAA during the first round of selection (Fig. 3A) and the fraction of HAA as a function of increasing selection rounds for different target concentrations (Fig. 3B). As the target concentration decreases, we found that HAA enrichment changes dramatically - over three orders of magnitude, from 6.6 to 1240– verifying that the use of low target concentrations can dramatically enhance selection efficiency.

From this analysis, it is tempting to conclude that minimal target concentration should be exclusively used experimentally. However, in practice, other considerations will likely constrain the practical lower limit. Two important predictions of our model are that (i) there is a limit to the maximum enrichment attainable, and (ii) once the target concentration becomes sufficiently small, the additional enhancement from further reduction of target concentration is rather modest. The maximum enrichment achievable is given by the ratio of the bulk dissociation constant 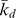
and the dissociation constant *k_d_* associated with HAA, 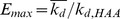. Once the target concentration drops below the *k_d_* of the HAA, further decreases in target concentration do not have a significant impact. For example, in decreasing the target concentration from 100 pM to 1 pM, we find that the enrichment of HAA increases only slightly, from 1212 to 1240 (Fig. 3A). In this case, the maximum enrichment factor is 1250, indicating that at target concentrations of 100 pM the enrichment is already very close to the theoretical maximum. In general, a useful rule of thumb for the experimentalist is to utilize target concentrations in the range of the *k_d_* of HAA.

### Modeling Enrichment with Background Binding

Another critical practical consideration is the fact that no experimental separation scheme can perfectly partition target-bound molecules from unbound and non-specifically bound background molecules. This is especially important when using extremely low target concentrations, as this background can overwhelm HAA population and undermine overall gains to enrichment. Mathematically, the enrichment of HAA in the presence of background aptamers (BA) can be expressed as
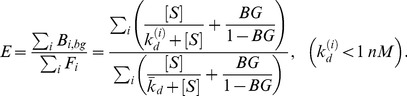
(23)where BG denotes the fraction of BA obtained during partitioning. This uses the results for 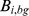 and 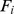 obtained in equation (19). We see that, in contrast to the model without background binding, HAA enrichment no longer increases monotonically as target concentration decreases. This is a consequence of the term involving BG. If the target concentration is too small, 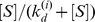 and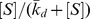 can become much smaller than 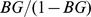, so that the enrichment of HAA becomes close to one–meaning no enrichment takes place. Therefore, BG limits the minimum target concentration and maximum enrichment that can be achieved during each round of selection. This makes intuitive sense, and suggests that for a given level of background aptamers, there exists an optimum target concentration that results in the most efficient selection of HAA.

### Optimal Target Concentration with Background Binding

**Figure 4:**
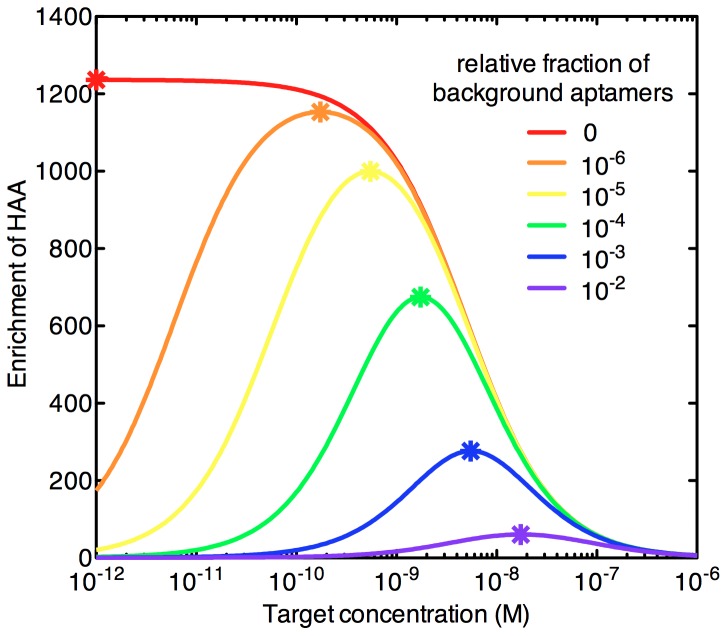
Effect of background partitioning on selection of high affinity aptamers. Enrichment of HAA during first rounds of selection is shown as a function of target concentration at different BG levels. The optimal target concentration for maximum enrichment of HAA is shown as (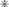). With no background, the lowest target concentration (1 pM) yields maximum HAA enrichment (1240-fold). As BG rises to 10^−6^, 10^−5^, 10^−4^, 10^−3^ and 10^−2^, this optimal target concentration increases to 174 pM, 550 pM, 1.74 nM, 5.5 nM and 17.4 nM respectively, yielding diminishing maximum HAA enrichment of 1153-, 999-, 674-, 276- and 60-fold, respectively.

Our model can be used to numerically calculate the target concentration that will achieve the maximum possible enrichment of HAA in the presence of background binding. To quantify the effect of BA on enrichment, we used our model to perform numerical simulations with different BG levels. For target concentrations ranging from 1 µM to 1 pM, we assumed BG ranging from 10^−6^ to 10^−2^, an experimentally reasonable range [13], [20], and calculated the enrichment of HAA using equation (23). The simulation results shown in Figure 4 reveal that when BG is taken into account, the enrichment of HAA differs markedly from the case without BG. As BG increases, maximum enrichment of HAA decreases and occurs at a higher target concentration. For example, when BG = 10^−6^, the optimal target concentration is 174 pM, and results in 1153-fold enrichment of HAA. On the other hand, when BG is increased to 10^−2^, the optimal target concentration becomes 17.4 nM, and yields significantly lower, 61-fold enrichment. Intuitively, this phenomenon can be explained by the fact that at lower BG, lower target concentrations can be applied during the selection without having the BA dominate the pool, leading to greater competition among aptamers to bind to the target. In this way, BG is an extremely important parameter that controls the SELEX process, and experimental methods should be optimized to keep its value to a minimum.

### Multiple Selection Rounds with Background Binding

To gain further insight into the importance of both target concentration and background during selection, we simulated multiple rounds of selection with two different experimental systems. The first mimics microfluidic selection (MF) using magnetic particles, which exhibit low background binding (BG∼10^−6^) with the capacity to handle very small amount of target molecules [14], [15], [18], [19]. The second system is modeled after nitrocellulose filter-based separation (NCF), which exhibits relatively high background binding, on the order of BG∼10^−2^ [14], [15], [16], [17]. Next, we simulated a complete SELEX experiment in which six rounds of selection were performed using either method. For each round of selection, we calculated HAA enrichment as a function of target concentration using equation (23) for both methods. Importantly, we used the optimal target concentration for each round, which was numerically calculated from the affinity distribution of the pool generated by the previous round of selection.

**Figure 5:**
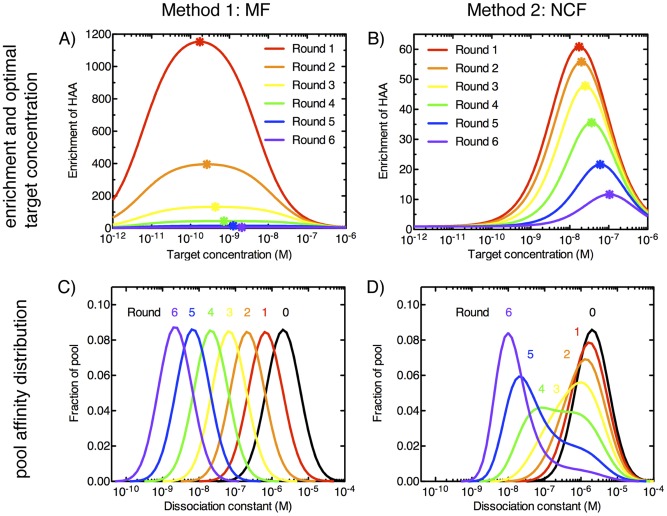
HAA enrichment during each round of selection as a function of target concentration. Two SELEX methods, microfluidic (A) or nitrocellulose filter-based SELEX (B) is shown. We calculated enrichment using equation (23). Optimal target concentration for achieving maximum enrichment is shown as (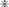). These optimal target concentrations were subsequently chosen for each round of SELEX to simulate the new affinity distribution of the selected pool for both microfluidic (C) and filter-based SELEX (D).

We note that the MF method yields significantly higher enrichment of HAA compared to the NCF method (Fig. 5A and B). For example, during the first round of selection, the MF method exhibits HAA enrichment of 1153-fold, ∼19 times greater than that achieved by NCF. Importantly, the MF method is also more robust when the target concentration deviates from the optimum, as indicated by shallower slope of the curve near the optimal concentration. For example, in the MF method, a 10-fold deviation in the optimal target concentration in the first round would result in a 20% decrease in the HAA enrichment from 1153- to 910-fold. However, such a deviation in the NCF method would result in a 60% decrease, reducing the HAA enrichment from 61- to 25-fold. In practice, this is a highly useful feature as it offers a significantly larger tolerance for experimental variability.

Over the course of multiple rounds of SELEX, the affinity distributions of the aptamer pools enriched via these two selection methods exhibit interesting differences. In the MF approach, we observe a uniform shift in affinity distribution from round to round, as BG plays a minimal role (Fig. 5C). On the other hand, interesting distortions in the affinity distribution can be observed in the NCF method, especially in rounds 3, 4 and 5 (Fig. 5D). This is a result of the greater impact of BG, which causes a slow shift in the aptamer population from the low affinity pool (right peak) to the high affinity pool (left peak).

**Figure 6:**
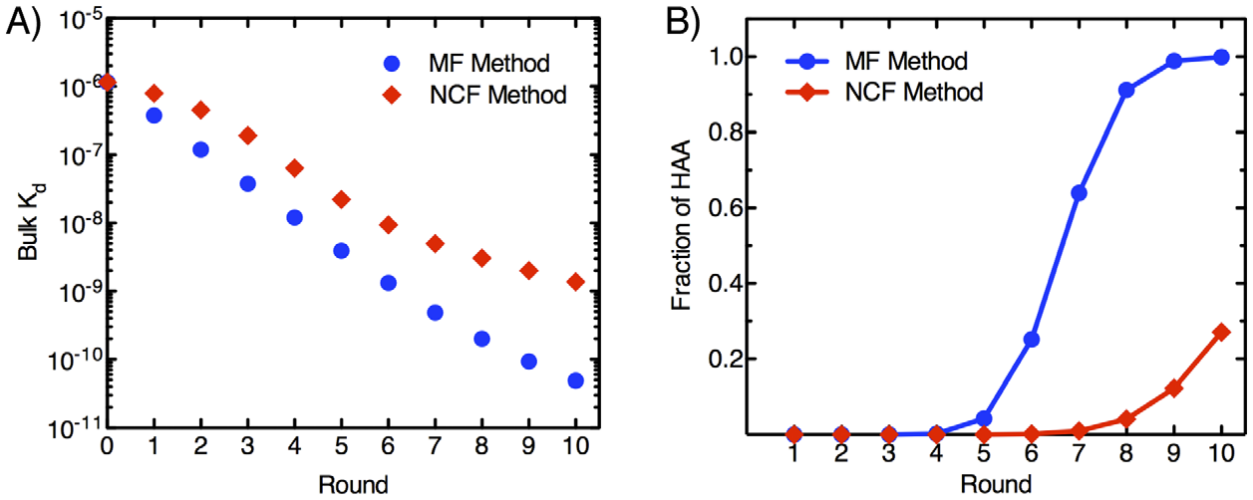
Properties of aptamer pools selected using NCF or MF methods. Ten rounds of selection with NCF or MF methods using optimal target concentrations for each round were simulated. (A) Bulk dissociation constant (*k_d_*) of selected pools after each round of selection. (B) Proportion of HAA at each round of selection. We assume BG of 10^−2^ and 10^−6^ for the MF and NCF methods, respectively.

Next, using Equation (10), we calculated the bulk 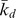 for 10 rounds of selection using the two methods. We observe that bulk 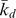 decreases at a significantly higher rate with MF compared to NCF, ultimately resulting in a pool that exhibits a ∼30-fold difference in bulk 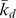 after ten rounds of simulated selection (Fig. 6A). Finally, we calculated the fraction of HAA after each round of selection for the two methods. Consistent with the findings shown in Fig. 6A, we note that the fraction of HAA increases at a markedly greater rate with MF method compared to NCF (Fig. 6B). For MF, the fraction of HAAs is 25% after 6 rounds, reaches 91% after 8 rounds and saturates at 100% in the 9^th^ round. On the other hand, NCF yields a 12% fraction of HAA in the 8^th^ round and only reaches 27% after 10 rounds of selection.

## Discussion

In this work, we have explored the effect of background binding and target concentration on multiple rounds of aptamer selection. We found that minimal target concentrations yield the most efficient selection if one ignores the contaminating effects of non-specifically bound background aptamers. However, this is unrealistic, as practically all methods exhibit background binding at various levels, which represents a competitive presence that can undermine the efficiency gains that would otherwise be achieved at very low target concentrations. Accordingly, we have incorporated this crucial factor into our method for determining selection conditions that will yield the greatest enrichment of high-affinity aptamers. Our model requires two input parameters: background binding, which can be easily obtained experimentally, and the initial bulk affinity distribution, which is commonly expressed as a log-normal distribution. With this information, our model can calculate the optimal target concentration and predict the affinity distribution of the enriched aptamer pool after each round.

We used our model to characterize two different selection methods: conventional nitrocellulose filter-based SELEX with high background binding, and microfluidic SELEX with low background binding. We found that the range of target concentrations that will yield near-optimal efficiency is heavily dependent on the level of background. This window is very narrow in selection conditions with high levels of background binding, such as NCF. In contrast, the low background binding associated with MF results can tolerate wider range of target concentrations, enabling greater flexibility in experimental design. This finding emphasizes the desirability of reducing background binding as much as possible, not only to achieve more stringent selection pressure by using lower target concentrations, but also to reduce the sensitivity of the convergence of SELEX to the experimental conditions. Our simulations showed that low-background conditions also generate higher-affinity aptamers in fewer rounds. After choosing optimal target concentrations for each round with the two methods, we observed that the bulk dissociation constant decreased at a significantly higher rate for MF, resulting in a pool with a bulk dissociation constant that was ∼30-fold lower than that achieved in the pool selected via NCF. Furthermore, six rounds of MF SELEX were sufficient to generate a pool containing a percentage of high-affinity aptamers that could only be achieved after ten rounds of NCF SELEX.

Another interesting result is that the optimal target concentration increases as the library becomes more refined. This finding indicates that even in cases where the optimal target concentration cannot be determined in practice, it is still desirable to use a lower target concentration during initial rounds of selection and then increase this concentration in later rounds. We note that the average dissociation constant of the enriched pool can be used as an effective guideline for the target concentration that should be used for successive rounds to help enhance the convergence of selection. In this way, our model provides useful experimental principles for designing SELEX experiments. However, we note that the models and analyses presented in this work make a few key assumptions. First, we have used log-normal distribution to describe the aptamer dissociation constants within the initial library. This assumption has been commonly used in previous work [13], [19], but to our knowledge has not been experimentally validated. We note that our model can be readily modified to accommodate a different affinity distribution. Second, we assumed a Langmuirian 1∶1 interaction between aptamer and target. In reality, multivalent interactions and cooperative binding can occur, whereby more than one aptamer type binds the target molecule or the binding of one aptamer affects the target’s molecular interactions with other aptamers. Finally, it is important to note that our model, as with most previous models [12], [13], [15], assumes equilibrium mass-action kinetics. To extend the model, may be desirable to introduce factors that break equilibrium at certain stages during selection to help accelerate the dissociation of weakly-bound aptamers, such as an active wash process.

### Conclusion

In this work, we have developed a model that enables experimentalists to determine selection conditions that will yield an optimally enriched aptamer pool after each round of a SELEX experiment based on two input parameters: background binding and initial bulk affinity distribution. While aptamers served as the main experimental system of our model, our approach can be applied with minor modification to virtually any biocombinatorial library, including phage display, cell surface display and mRNA display. The critical influence of target concentration and background binding in these display technologies would be equally important, and in experimental practice, similar trade-offs would be required to achieve ideal selection efficiencies.
